# Radiolabeled Cationic Peptides for Targeted Imaging of Infection

**DOI:** 10.1155/2019/3149249

**Published:** 2019-10-29

**Authors:** Tolulope A. Aweda, Zumrut F. B. Muftuler, Adriana V. F. Massicano, Dhruval Gadhia, Kelly A. McCarthy, Stacy Queern, Anupam Bandyopadhyay, Jianmin Gao, Suzanne E. Lapi

**Affiliations:** ^1^Department of Radiology, University of Alabama at Birmingham, Birmingham, AL, USA; ^2^Institute of Nuclear Sciences, The University of Ege at Izmir, Izmir, Turkey; ^3^Department of Chemistry, Boston College, Chestnut Hill, Newton, MA 02467, USA; ^4^Department of Chemistry, Indian Institute of Technology, Ropar, Punjab, India

## Abstract

Molecular probes targeting bacteria provide opportunities to target bacterial infections in vivo for both imaging and therapy. In the current study, we report the development of positron emission tomography (PET) probes for imaging of live bacterial infection based on the small molecules HLys-DOTA, a polycationic peptide synthesized as the D-isomer (RYWVAWRNRG) conjugated to 1, 4, 7, 10-tetraazacyclododecane-N′,N″,N‴,N-tetraacetic acid (DOTA) and AB1-HLys-DOTA, which includes an unnatural amino acid AB1 that preferentially binds to bacteria membrane lipids with amine groups via formation of iminoboronates. HLys-DOTA and AB1-HLys-DOTA peptides were radiolabeled with ^64^Cu and investigated as PET imaging agents to track bacterial infection in vitro and in intramuscularly infected (IM) mice models. Cell uptake studies at 37°C in *Staphylococcus aureus* (SA) show higher uptake of ^64^Cu-AB1-HLys-DOTA; 98.47 ± 3.54% vs ^64^Cu-HLys-DOTA; 39.12 ± 3.27% at 24 h. Standard uptake values (SUV) analysis of the PET images resulted in mean SUV of 0.70 ± 0.08, 0.49 ± 0.04, and 0.31 ± 0.01 for ^64^Cu-AB1-HLys-DOTA and 0.17 ± 0.06, 0.16 ± 0.02, and 0.13 ± 0.01 for ^64^Cu-HLys-DOTA at 1, 4, and 24 h post injection, respectively, in the infected muscles. Similarly, in the biodistribution studies, dose uptake in the infected muscles was 4 times higher in the targeted ^64^Cu-AB1-HLys-DOTA group than in the ^64^Cu-HLys-DOTA group and 2‐3 times higher than in the PBS control group at 1, 4, and 24 h post injection. ^64^Cu-AB1-HLys-DOTA was able to distinguish between SA-infected muscle and *Pseudomonas aeruginosa* (PA) infected muscle with lower mean SUV of 0.28 ± 0.10 at 1 h post injection. This illustrates the utility of the AB1 covalently targeting group in synergy with the HLys peptide, which noncovalently binds to bacterial membranes. These results suggest that ^64^Cu-labeled AB1-HLys-DOTA peptide could be used as an imaging probe for detection of bacterial infection in vivo with specificity for Gram-positive bacteria.

## 1. Introduction

Infectious diseases are one of the top ten leading causes of death in the world and the number one cause of death in low-income developing countries. Current diagnostic methods for bacterial infections are time-consuming as they require samples from the patient to be cultured for hours to weeks before enough bacteria are isolated to run destructive diagnostic tests [1, 2]. As proper identification of the disease often requires long diagnostic processes and bacterial infections tend to progress rapidly, fast diagnostic techniques would allow physicians to identify what types of antimicrobial therapy may help a patient in a clinically relevant timeframe. The ability to image bacterial disease would permit rapid and specific diagnosis of infection. In addition, the ability to image infections would allow clinicians to determine whether a therapy is effective and to monitor patient response to therapy.

Recently, there has been an increase in research into noninvasive imaging of bacterial infection using PET/CT, SPECT, MRI, and other imaging modalities in preclinical studies of infectious diseases using natural and synthetic molecules [3]. The use of radionuclides for detecting and localizing infection has been employed for decades [4, 5]. Ideally, the radiopharmaceutical for detecting infection should be specific, sensitive, and clear from the body rapidly to facilitate early image acquisition and clear delineation of the infected area. These radionuclides have been utilized as salts or small molecules such as ^99m^Tc-methylene diphosphonate, ^67^Ga-citrate, and ^18^F-FDG [6–10]. ^111^In-oxine and ^99m^Tc-exametazime have also been used to label leukocytes for imaging in diagnosis of infection [7, 11, 12]. This approach, though valuable, has some drawbacks, requiring high levels of leukocytes, and detection of noninfectious inflammations and is thus not specific to the infection or bacteria [13, 14].

Another radioisotope of interest is ^68^Ga, with a shorter half-life of 68 min compared to its counterpart ^67^Ga: half-life of 78.3 h (used in SPECT), has undergone an increased utilization in preclinical and clinical PET imaging particularly in oncology. Although, [^68^Ga]citrate PET-CT of bone and joint infection offered lower radiation dose, as well as earlier and shorter imaging times, and its sensitivity and specificity was found to be lower than [^67^Ga ]citrate [15]. In other infection and inflammation imaging studies, ^68^Ga has been used to radiolabel ligands, small molecules, and peptides to target a host of receptors, inhibitors, leukocytes, and pathways [16]. ^68^Ga-apo-transferrin ([^68^Ga]TF) has been used to image *Staphylococcus aureus* (SA) and found to detect the infected lesions better than [^68^Ga]Cl_3_; additionally, [^68^Ga]TF was also found to detect the Gram-negative bacteria, *Proteus mirabilis* [17]. In other studies, the antibiotic ciprofloxacin was labeled with ^68^Ga and used to distinguish inflamed muscle from SA-infected muscle [18].

The use of ^64^Cu radioisotope for imaging purposes has increased over the years, and several studies have utilized this isotope to label compounds for detecting regions of infection [19]. In order to label these compounds, several suitable chelators for ^64^Cu have been developed with significant progress over the years; these chelates range from acyclic to cage-like bifunctional chelators [20, 21]. ^64^Cu, with its relatively long half-life (12.7 hours) compared to ^18^F or ^99m^Tc has been used to label peptides, antibody fragments, and whole antibodies for imaging [22]. For instance, necrotic pulmonary tuberculosis lesions in chronically infected mice were detected and demonstrated to be hypoxic using ^64^Cu(II)-diacetyl-bis(N^4^-methyl-thiosemicarbazone), [^64^Cu]ATSM [23]. In mice models of *S. aureus* endocarditis (heart valve infection), robust localization of [^64^Cu]DTPA-prothrombin was used to noninvasively detect infection lesions as compared to the bacteria-free control mice, which had no accumulation at the site of endothelial trauma [24].

**Figure 1:**
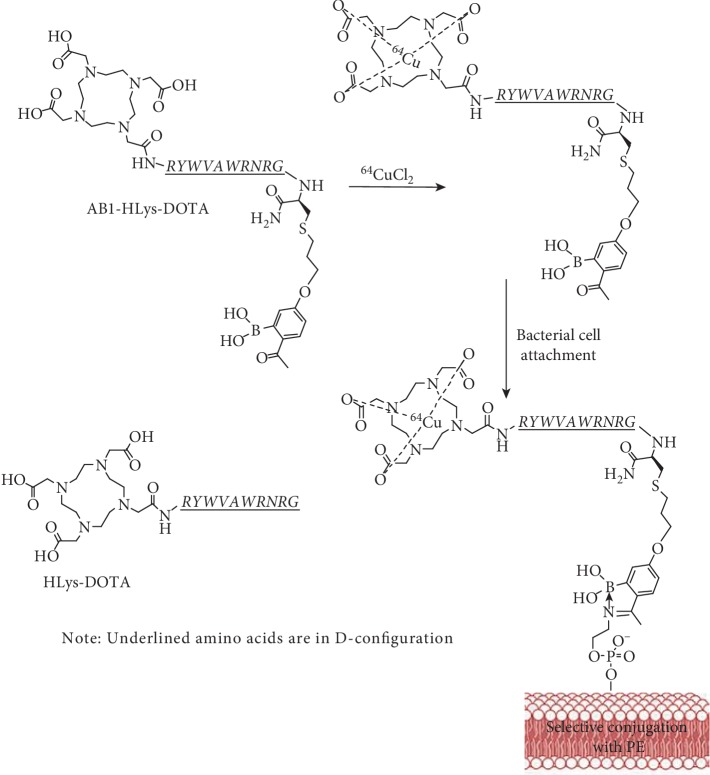
Illustration of ^64^Cu-radiolabeled AB1-HLys-DOTA peptide for targeting PE on bacterial cell surfaces.

Radiolabeled molecular probes specifically targeting bacterial lipids provide opportunities to target bacterial infections in vivo for both imaging and therapy. In this study, synthetic peptides that preferentially bind to bacterial surfaces with amine-presenting lipids were radiolabeled and studied in vitro and in vivo. Two phospholipids abundant on Gram-positive bacteria, phosphatidylethanolamine (PE) and lysylphosphatidylglycerol (Lys-PG), were targeted for recognition by appropriately constructed short peptides used as low molecular weight probes [25]. For our studies, the D-isomer of a polycationic peptide HLys (RYWVAWRNRG), which binds noncovalently to bacteria surfaces, was conjugated to 1, 4, 7, 10-tetraazacyclododecane-N′, N″, N‴, N-tetraacetic acid (DOTA) to make HLys-DOTA. To improve the binding of the peptide to the bacterial surface, an unnatural amino acid AB1, which preferentially binds covalently to lipids with amine groups via formation of iminoboronates under physiological conditions, was conjugated to HLys-DOTA, as shown in Figure 1 [26]. By targeting PE and Lys-PG with AB1, the iminoboronate chemistry allows potent labeling of Gram-positive bacteria such as *Staphylococcus aureus* (SA) even in the presence of serum proteins, while bypassing mammalian cells and Gram-negative bacteria [26]. HLys-DOTA and AB1-HLys-DOTA peptide conjugates were radiolabeled with ^64^Cu and studied as PET imaging agents to track bacterial infection in vitro and in intramuscularly infected (IM) mice models.

## 2. Materials and Methods

^64^Cu was produced in-house on an ACSI TR-24 Cyclotron (Richmond, Canada) located in the UAB Cyclotron facility. 2-deoxy-2-[^18^F]fluoro-d-glucose ([^18^F]FDG) was purchased from PETNET (Birmingham, AL). All solvents and reagents were obtained from Sigma-Aldrich Company unless otherwise specified. *Staphylococcus aureus* (SA), ATCC 6538, and *Pseudomonas aeruginosa* (PA), ATCC 47085, were purchased from ATCC (Manassas, VA), and Whatman 3 M silica gel thin-layer chromatography (TLC) plates were purchased from Fisher Scientific (Pittsburgh, PA). Diethylenetriaminepentaacetic acid (DTPA) was purchased from Sigma (St. Louis, MO). 1,4,7,10-Tetraazacyclododecane-1,4,7,10-tetraacetic acid mono-N-hydroxysuccinimide ester (DOTA-NHS-ester) was purchased from Macrocyclics (Dallas, Texas). C18 SepPak cartridges were obtained from Waters Corporation (Milford. MA). Radiometric instant thin-layer chromatography measurements were accomplished using a TLC scanner (Bioscan AR-2000 Scanner). Reverse-phase chromatography analysis was performed on a C18 column (PA) with an infinity diode-array UV detector (Agilent, Lake Forest, CA) and a PMT/NaI remote radioactive detector (LabLogic Systems Ltd, Brandon, FL). Laura radiochromatography software (LabLogic Systems Ltd, Brandon, FL) was used to quantify chromatograms by integration. Radioactive samples were counted using a 2480 Wizard II automatic gamma counter (PerkinElmer, Downers Grove, IL). Imaging studies were done by using GNEXT PET-CT (Sofie BioSciences) [CT-80 Kvp, 5 min (approximately 100 *μ*m), PET-Resolution ≤1 mm]. Acquired PET/CT images were analyzed with Inveon™ Research Workplace (IRW) (Siemens).

### 2.1. Synthesis and Structural Analysis of HLys-DOTA and AB1-HLys-DOTA Peptides

HLys-DOTA and AB1-HLys-DOTA were synthesized by following a previously described protocol for solid-phase peptide synthesis [26], and the DOTA moiety was conjugated to the N-terminus of peptides on resin using DOTA-NHS-ester as a precursor [27]. To ensure in vivo stability of the peptides, the D-isomer of all amino acids except AB1 was used for peptide synthesis. Literature reports have shown that the D-isomers of antimicrobial peptides behave similarly to their L-counterparts in bacterial binding [28]. The peptides were purified using HPLC, and their purity and integrity was confirmed by LC-MS (Supporting Information [Supplementary-material supplementary-material-1]).

### 2.2. Production of ^64^Cu

^64^Cu (*t*_1/2_ = 12.7 h, *β*^+^ = 17%, *β*^−^ = 39%, EC = 43%, *E*_max_ = 0.656 MeV) was produced in-house at the University of Alabama at Birmingham Cyclotron facility via the ^64^Ni(p,n)^64^Cu nuclear reaction. Production and purification of ^64^Cu was conducted using modified methods from literature [29, 30] via bombardment of enriched ^64^Ni targets at 40 *μ*A with an incident proton beam of 19 MeV degraded to 12 MeV with a 1 mm Al degrader.

### 2.3. Radiolabeling of HLys-DOTA and AB1-HLys-DOTA Peptides

The two peptides, HLys-DOTA and AB1-HLys-DOTA, were radiolabeled with ^64^CuCl_2_ buffered in 0.1 M NH_4_OAc, pH 6, at 56°C according to modified previously published methods [31, 32]. Briefly, radiolabeling of the peptides was achieved by adding 50 *μ*g of HLys-DOTA or AB1-HLys-DOTA into 120–130 MBq (3.2–3.5 mCi) of ^64^CuCl_2_ in 1450 *μ*L of 0.1 M NH_4_OAc, pH 6. The reactions were incubated on a mixer with 800 rpm agitation at 56°C for 45 min, and radiolabeling was also attempted at lower temperature. Radiolabeling yield and radiochemical purity were assessed using silica gel plates developed in 50 : 50 of methanol: 1 M ammonium acetate and high-performance liquid chromatography, respectively, with gradient: 0–12 min: 95% A to 20% A (A = water, 0.1% TFA, B = acetonitrile, 0.1% TFA), 12–15 min: 20% A to 95% A. ^64^Cu-AB1-HLys-DOTA was purified according to the Jacobson method [33]. A SepPak C18-cartridge was activated with 5 mL of ethanol and 10 mL of water. The reaction mixture of ^64^Cu-AB1-HLys-DOTA was diluted with 5 mL of water and loaded slowly onto the activated SepPak C18-cartridge using a syringe. The cartridge was washed with 10 mL of water followed by elution of the desired labeled peptide with 1 mL of 10 mM HCl in ethanol. The ethanol was evaporated, and the radiolabeled peptide was reformulated with saline.

### 2.4. Serum Stability Studies

An aliquot of 10 *μ*L of 3.3 MBq (∼90 *μ*Ci) of ^64^Cu-labeled AB1-HLys-DOTA compound was added to 90 mL of PBS, human serum (HSA), or mouse serum and incubated at 37°C with agitation (500 rpm) separately. Aliquots were removed at time points 0, 0.17, 1, 2, 4, and 24 h and analyzed using radio-TLC. All reactions were conducted in triplicate.

### 2.5. In Vitro Studies

#### 2.5.1. Bacterial Cell Culture

Bacterial in vitro uptake and imaging experiments were performed using Gram-positive bacteria, *Staphylococcus aureus* (SA), and Gram-negative bacteria, *Pseudomonas aeruginosa* (PA), as control. Bacterial cells from a single colony were grown overnight in LB broth at 37°C with agitation until the cells reached the mid logarithmic phase (OD_600_∼0.6–0.7). For the in vitro experiments, 750–1000 *μ*L of the bacterial cell culture was spun down at 7000 rpm for 7 min in a 1.5 mL centrifuge tube.

#### 2.5.2. Uptake Studies of ^64^Cu-Labeled Peptides in *Staphylococcus aureus* (SA) and *Pseudomonas aeruginosa* (PA)

Bacteria cell uptake studies of ^64^Cu-HLys-DOTA and ^64^Cu-AB1-HLys-DOTA in SA and PA bacteria were performed at 37°C. LB broth was inoculated with SA or PA bacteria and after 18–22 hours, cells were harvested by spinning down and washing twice in 750–1000 *μ*L of PBS (50 mM sodium phosphate, pH = 7.4), and the OD_600_, OD_230_, and OD_260_ determined. 750 *μ*L of the PBS suspended bacteria (0.9–1.3 × 10^7^ CFU/mL) was incubated with 0.925–0.999 MBq (25–27 *μ*Ci) of ^64^Cu-HLys-DOTA and ^64^Cu-AB1-HLys-DOTA at 37°C in triplicates at different time points: 5, 40, 60, 240, and 1440 min. At each time point, the mixture was spun down, washed twice with PBS, and the final bacterial pellets were measured using a gamma counter to determine the percent of associated radiolabeled peptide. Mammalian cell uptake was verified by following a similar procedure in a nonbacterial cell, SKBR3 breast cancer cell line.

#### 2.5.3. Small Animal Imaging and Biodistribution Studies

All animal experiments were performed according to animal use protocols approved by the University of Alabama at Birmingham Institutional Animal Care and Use Committee (IACUC). Animals were housed under controlled conditions with a natural light-dark cycle. They were allowed to adapt to the housing environment for at least 48 h prior to study. Biodistribution and PET imaging studies with ^64^Cu-HLys-DOTA and ^64^Cu-AB1-HLys-DOTA were conducted in mice intramuscularly (IM) infected in the thigh muscle with SA and PBS as a control. Thirty-six male mice (25–30 g, 4–5 weeks old) infected with SA were used for animal studies. For each peptide, 4 mice were used for small animal PET imaging at 1, 4, and 24 h, and then sacrificed at 24 h for a post-PET biodistribution. Another set of 4 mice each was used for biodistribution studies at 1 and 4 h. A similar set of mice was used for blocking studies with ^64^Cu-AB1-HLys-DOTA peptide. In order to check the specificity of ^64^Cu-AB1-HLys-DOTA for Gram-positive bacteria, imaging and post-PET biodistribution studies were conducted in another set of mice (four) intramuscularly (IM) infected in the thigh muscle with PA and PBS.

#### 2.5.4. Infection Animal Model

Mice for infection studies were anesthetized prior to the intramuscular infection of SA or PA. Animals were immobilized with isoflurane. Bacteria cells concentrated in sterile PBS were injected into the thigh muscle and the site of injection pinched to minimize bleeding. SA or PA suspension (50 *μ*L, 2.6–2.8 × 10^7^ CFU per mouse) was intramuscularly injected into the right thigh muscle, and sterile PBS (50 *μ*L) was injected into the left thigh muscle as control. Mice were placed back into the cages and allowed to recover with frequent monitoring to identify any sign of extreme distress. The infection was allowed to develop for 3–5 days before intravenous injection of the radiolabeled probe. The muscles on the left and right thighs were harvested, homogenized, and plated on mannitol agar plates or cultured in LB broth to verify the presence of infection. In addition, [^18^F]FDG imaging was also performed in infected animal models as described in the PET imaging section below to further confirm the existence of infection in the right thigh and the absence thereof in the left thigh.

#### 2.5.5. Biodistribution Studies

For biodistribution studies, 3.3–3.7 MBq (90–100 *μ*Ci) of ^64^Cu-HLys-DOTA or ^64^Cu-AB1-HLys-DOTA was injected into prewarmed tail vein of the animals under anesthesia. Animals were euthanized at 1 and 4 h after radiotracer administration (*n* = 4 for each tracer at each time point). At 24 h after small animal PET imaging, the mice were also euthanized for a post-PET biodistribution. In a similar set of experiment, excess of the unlabeled peptide, 150 nmol, was coinjected with the radiolabeled peptides per mouse. The organs of interest were collected, weighed, and measured for radioactivity content using a gamma counter. The data were background-corrected and decay-corrected. The biodistribution data were expressed as percentage of injected radioactive dose per gram of tissue (%ID/g) for selected organs as the mean value of four mice.

#### 2.5.6. PET/CT Imaging Studies

Small animal PET/CT imaging studies were conducted in SA-infected and PA-infected animal models. Each radiolabeled peptide, 3.3–3.7 MBq (90–100 *μ*Ci), was administrated via tail vein injection of prewarmed mice under anesthesia. The inflammatory response caused by the infection was confirmed by intravenous injection of [^18^F]FDG, 3.7 MBq (100 *μ*Ci), into mice infected on the right thigh muscle with SA or PA and on the left with PBS as control. For this small animal PET imaging, 1 and 4 h post-injection static scans were collected. Following imaging studies, the mice were sacrificed for biodistribution. Mice were allowed to recover from anesthesia until the time of imaging. Mice were anesthetized with 2‐3% isoflurane/oxygen and imaged on the small animal PET/CT scanner. Static images were collected at 1, 4, and 24 h for 15, 20, and 30 min, respectively. PET images were coregistered with CT image for anatomical colocalization. Regions of interest (ROI) were manually drawn over organs of interest with CT anatomical guidelines, and the associated radioactivity was measured using Inveon Research Workstation software. Standard uptake values (SUV) were calculated as nCi/cc × animal weight/injected dose, and comparisons in pharmacokinetics of radiolabeled peptides were assessed.

### 2.6. Statistical Analysis

Statistical calculations were carried out using Prism 7 (GraphPad Software) and expressed as mean ± SD. One-way analysis of standard deviation at 95% confidence level (*p* < 0.05) were considered statistically significant.

## 3. Results

### 3.1. HLys-DOTA and AB1-HLys-DOTA Peptide Synthesis, Characterization, and Stability

The analysis of HLys-DOTA and AB1-HLys-DOTA peptides was performed using LC-MS in positive mode [M+H] allowing detection of their corresponding molecular ion at *m*/*z* 1691.92 and *m*/*z* 2074.15, respectively [26]. HLys-DOTA and AB1-HLys-DOTA peptides were radiolabeled with ^64^Cu at molar activities of 3873 ± 398 and 3809 ± 943 MBq/*μ*mol (105 ± 11 and 103 ± 25 mCi/*μ*mol), respectively, at 56°C. Incubations at lower temperature resulted in lower yields (See Supporting Information [Supplementary-material supplementary-material-1] for radiolabeling kinetics). Radiochemical and labeling yield of ^64^Cu-HLys-DOTA was ≥95% after TLC and HPLC analysis but ^64^Cu-AB1-HLys-DOTA required purification through a C18 SepPak to achieve ≥95% radiochemical purity. The need for extra purification of ^64^Cu-AB1-HLys-DOTA in order to achieve ≥95% radiochemical purity could be due to the extra benzyl group and the hydrophobic portion of AB1 preventing good complexation of radiocopper ions.

### 3.2. Stability and In Vitro Cell Uptake Studies

**Figure 2:**
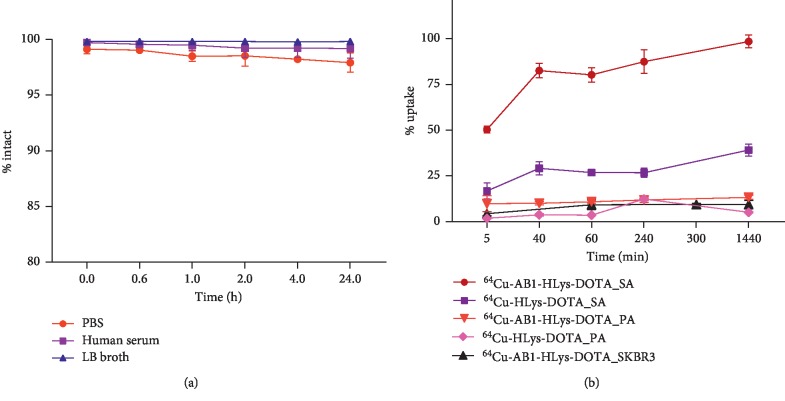
Stability of (a) ^64^Cu-AB1-HLys-DOTA in PBS, human serum, and LB broth at 0–24 h and 37°C. (b) Cell uptake of ^64^Cu-AB1-HLys-DOTA and ^64^Cu-HLys-DOTA peptides in *S. aureus* (SA) and *P. aeruginosa* (PA), and ^64^Cu-AB1-HLys-DOTA in mammalian SKBR3 cells.

Excellent stability of ^64^Cu-AB1-HLys-DOTA was observed in the PBS, human serum, and LB broth with >98% intact peptide observed at 24 h post incubation in all solutions, as shown in Figure 2(a). Bacteria cell uptake of ^64^Cu-AB1-HLys-DOTA conjugate at 37°C in *Staphylococcus aureus* (SA) was significantly higher than that of ^64^Cu-HLys-DOTA at all time points: 0.08, 0.67, 1, 4, and 24 h; 98.5 ± 3.5% vs 39.1 ± 3.3% at 24 h, Figure 2(b). Specific uptake in Gram-positive bacteria SA was confirmed by the lower uptake of the peptides in *Pseudomonas aeruginosa* (PA), a Gram-negative bacterium. At 5 min after incubation, the uptake of ^64^Cu-AB1-HLys-DOTA and ^64^Cu-HLys-DOTA showed 9.8 ± 4.4% and 1.9 ± 0.4% binding to PA, respectively, which increased slightly to 13.3 ± 1.1% and 5.2 ± 0.3%, respectively, at 24 h after incubation. This indicates a 5–10 times lower uptake of the peptides in PA as compared to SA confirming the specificity of AB1 for Gram-positive bacteria. Specific bacterial cell uptake was confirmed by the low uptake (9.4 ± 2.3%) of ^64^Cu-AB1-HLys-DOTA in mammalian SKBR3 breast cancer cells at 24 h post incubation.

### 3.3. Confirmation of Infection in Animal Model

The development of infection was evident by presence of a palpable mass filled with abscess observed during dissection and harvesting. The infected muscle and control muscle were harvested, homogenized, and the CFU of bacteria counted. In the infected muscle, 4.8 ± 2.5 × 10^6^ cfu/g of bacteria was found and only one PBS-control muscle was positive with colony at 1.8 × 10^5^ cfu/g cfu. [^18^F]FDG in vivo characterization of the infection site verified high uptake in the SA-infected muscle versus the control muscle at 1 and 4 h post injection, *p* < 0.005.

### 3.4. Biodistribution Studies

**Figure 3:**
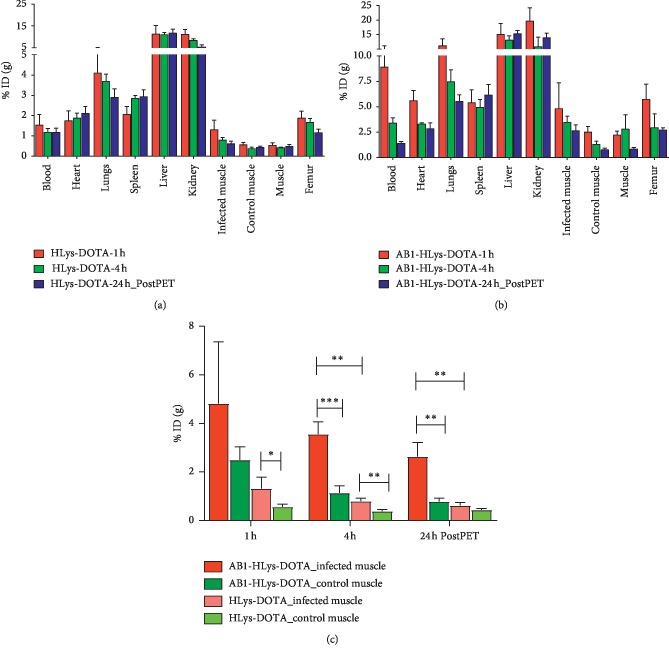
Biodistribution of (a) ^64^Cu-HLys-DOTA and (b) ^64^Cu-AB1-HLys-DOTA in intramuscularly infected mice model at 1, 4, and 24 h post injection. (c) Comparison of dose uptake of ^64^Cu-HLys-DOTA and ^64^Cu-AB1-HLys-DOTA in the infected and PBS-injected control muscle. *n* = 4 for each time point, ^*∗*^, ^*∗∗*^, and ^*∗∗∗*^ show significant statistical analysis at *p* < 0.05.

The biodistribution and pharmacokinetics of the peptides ^64^Cu-AB1-HLys-DOTA and ^64^Cu-HLys-DOTA were assessed in mice intramuscularly infected with *S. aureus* on the right thigh muscle and PBS injected in the left thigh muscle as control. The biodistribution showed that the uptake of both peptides in the infected muscle was significantly higher than the PBS-injected control muscle of the same mouse at all time points: 1, 4, and 24 h post injection, as shown in Figure 3. The uptake of ^64^Cu-AB1-HLys-DOTA conjugate in the infected thigh was 2‐3 times higher than the control muscle at later time points, although no significant difference was observed at 1 h (4.8 ± 2.5 %ID/g vs. 2.5 ± 0.5 %ID/g). At 4 h, the dose uptake of ^64^Cu-AB1-HLys-DOTA in the infected muscle was 3.6 ± 0.5 %ID/g vs. 1.1 ± 0.4 %ID/g in the control muscle, *p* < 0.0005. Similarly, at 24 h, the uptake of ^64^Cu-AB1-HLys-DOTA in the infected muscle was 2.6 ± 0.6 %ID/g vs. 0.8 ± 0.1 %ID/g in the control muscle, *p* < 0.005. In contrast, the dose accumulation of ^64^Cu-HLys-DOTA in the infected muscle was only 1- or 2-fold higher than the control muscle in the same mouse, except at 24 h where no significant difference was observed between them. At 1 and 4 h, the uptake of ^64^Cu-HLys-DOTA in the infected muscle vs. control muscle was 1.3 ± 0.5 %ID/g vs. 0.6 ± 0.1 %ID/g, *p* < 0.05, and 0.8 ± 0.1 %ID/g vs. 0.4 ± 0.1 %ID/g, *p* < 0.005, respectively. We also observed that uptake in the infected muscle was 4 times higher in the targeted ^64^Cu-AB1-HLys-DOTA group compared to that in the ^64^Cu-HLys-DOTA group, as shown in Figure 3(c). The uptake of ^64^Cu-AB1-HLys-DOTA in the infected muscle was 4.8 ± 2.5, 3.6 ± 0.5, and 2.6 ± 0.6 %ID/g vs. 1.3 ± 0.5, 0.8 ± 0.1, and 0.6 ± 0.1 %ID/g for ^64^Cu-HLys-DOTA, respectively, at 1, 4, and 24 h post injection, *p* < 0.005 at 4 and 24 h. The highest accumulation of the radiolabeled peptides was observed in the liver and kidney with ^64^Cu-AB1-HLys-DOTA also showing some uptake in the lungs. ^64^Cu-AB1-HLys-DOTA also showed higher retention in the blood and heart than ^64^Cu-HLys-DOTA at 1 and 4 h post injection but at 24 h, both peptides had similar activity in the blood. A similar experiment using an excess of the unlabeled peptides, 150 nmol, did not show a statistical difference (data not shown) in the uptake of the peptides at the infection site. This may indicate that the binding sites at the bacterial surface cannot be saturated.

### 3.5. PET/CT Imaging

**Figure 4:**
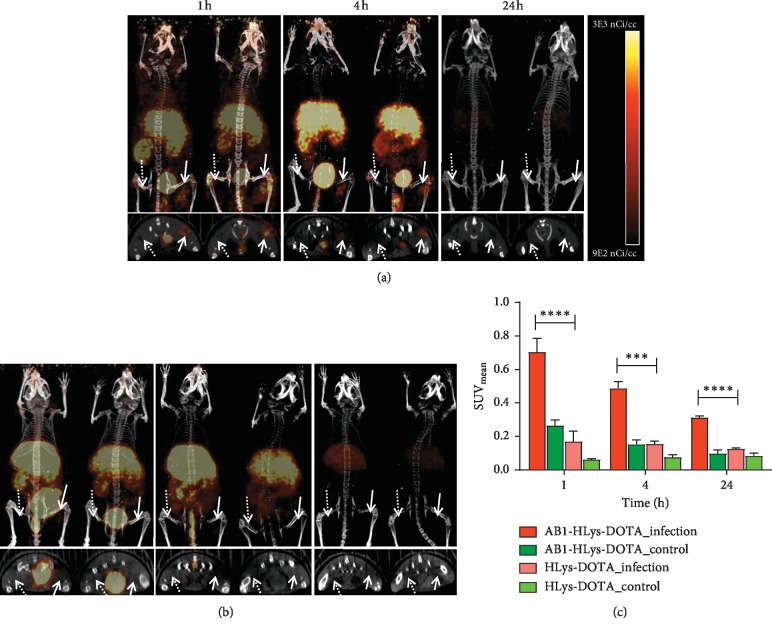
Small animal PET/CT images of (a) ^64^Cu-HLys-DOTA, (b) ^64^Cu-AB1-HLys-DOTA, and (c) mean SUV values calculated from infected and PBS-control muscle. Dashed arrow indicates the PBS-control muscle, and solid arrow indicates the infected right muscle. *n* = 4 for each time point, ^*∗∗∗*^ and ^*∗∗∗∗*^ show significant statistical analysis at *p* < 0.0005 and *p* < 0.0001, respectively.

The uptake of ^64^Cu-AB1-HLys-DOTA in the infected muscle was visibly observed via small animal PET/CT imaging as early as 1 and 4 h post injection, Figure 4. The uptake in the infected muscle was higher in the PET images of ^64^Cu-AB1-HLys-DOTA than in the images of ^64^Cu-HLys-DOTA at 1 and 4 h post injection in agreement with the biodistribution data. SUV analysis of the PET images showed 2.4 to 4-fold increase in accumulation of ^64^Cu-AB1-HLys-DOTA vs. ^64^Cu-HLys-DOTA at the infection. The mean SUV of ^64^Cu-AB1-HLys-DOTA and ^64^Cu-HLys-DOTA in the infected muscle was 0.70 ± 0.08 vs. 0.17 ± 0.06, *p* < 0.0001, 0.49 ± 0.04 vs. 0.16 ± 0.02, *p* < 0.0005, and 0.31 ± 0.01 vs. 0.13 ± 0.01, *p* < 0.0001 at 1, 4, and 24 h post injection, respectively. There was no statistical difference between mean SUV uptake of ^64^Cu-HLys-DOTA in the infected muscle and ^64^Cu-AB1-HLys-DOTA uptake in the control muscles at all time points. This indicates ^64^Cu-HLys-DOTA uptake in the infected muscle is as low as the background uptake of ^64^Cu-AB1-HLys-DOTA observed in the control muscle. Mean SUV analysis of the heart supports the trend in the biodistribution study with blood retention of ^64^Cu-AB1-HLys-DOTA at 0.65 ± 0.08 vs. 0.25 ± 0.05 for ^64^Cu-HLys-DOTA, *p* < 0.0005, at 1 h. At 24 h post injection, both peptides had similar dose retention in the heart: 0.26 ± 0.04 and 0.21 ± 0.04 for ^64^Cu-AB1-HLys-DOTA and ^64^Cu-HLys-DOTA, respectively. The images also showed high signal in the liver and kidney possibly due to the fast clearance of the cationic peptides through these organs.

Specificity of ^64^Cu-AB1-HLys-DOTA peptide for Gram-positive bacteria was confirmed by low uptake in the Gram-negative bacteria *Pseudomonas aeruginosa* (PA) as observed in the PET images and the 4 h post-PET biodistribution, shown in Supporting Information Figure 3. ^64^Cu-AB1-HLys-DOTA uptake in the SA-infected muscle at 1 and 4 h was significantly higher than that in the PA-infected muscle: 0.70 ± 0.08 vs. 0.28 ± 0.10, *p*=0.0052, and 0.49 ± 0.04 vs. 0.19 ± 0.10, *p*=0.0288, respectively. A similar trend was observed in the 24 h post-PET biodistribution with percent uptake of ^64^Cu-AB1-HLys-DOTA in the SA-infected muscle at 2.64 ± 0.57 %ID/g vs. 0.49 ± 0.11 %ID/g in the PA-infected muscle, *p*=0.0039.

**Figure 5:**
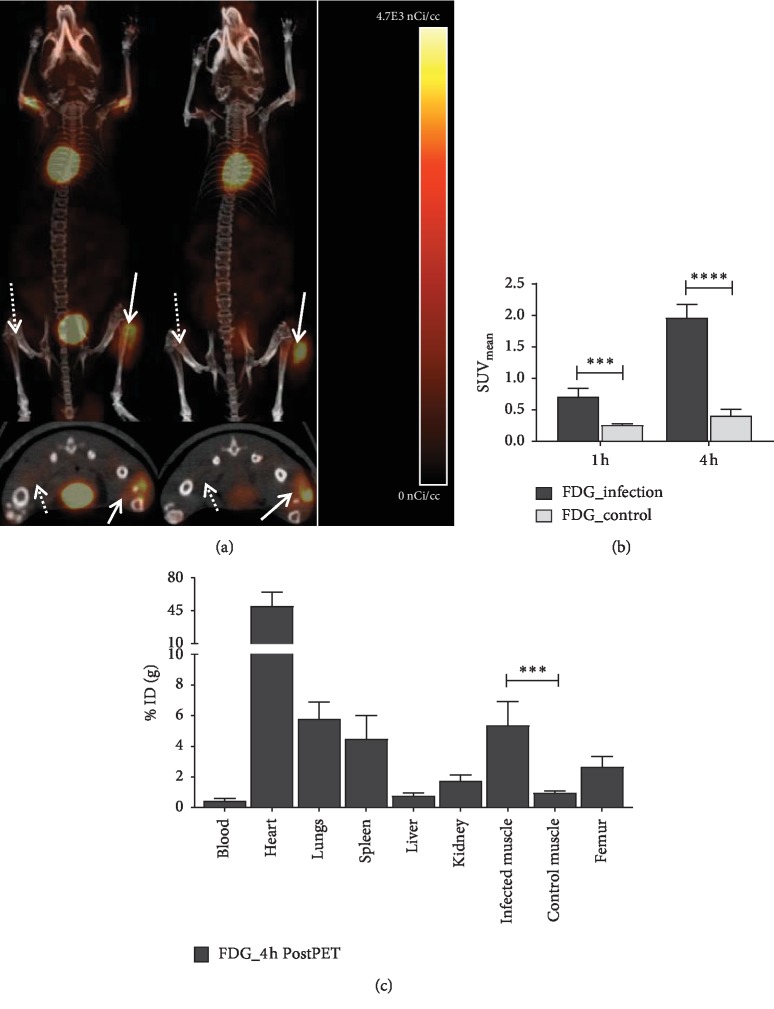
Small animal PET/CT images of (a) [^18^F]FDG, 4 h post injection, (b) mean SUV values calculated from infected and PBS-control muscle, and (c) post-PET biodistribution of [^18^F]FDG at 4 h after i.v. injection. Dashed arrow indicates the PBS-control muscle, and solid arrow indicates the infected right muscle. *n* = 6, ^*∗∗∗*^ and ^*∗∗∗∗*^ show significant statistical analysis at *p* < 0.0005 and *p* < 0.0001, respectively.

[^18^F]FDG uptake in the SA-infected muscle was significantly higher than that in the control muscle at 4 h post injection with mean SUV of 1.96 ± 0.19 vs. 0.41 ± 0.09, *p* < 0.0001, which confirmed the presence of the SA infection [34–36], as shown in Figures 5(a) and 5(b). The higher uptake of [^18^F]FDG in the SA-infected muscles was also confirmed with post-PET imaging biodistribution data at 4 h (5.4 ± 1.3 %ID/g vs. 1.0 ± 0.1 %ID/g, *p*=0.0009), as shown in Figure 5(c).

## 4. Discussion

Antimicrobial peptides produced by phagocytes and other cell types in the body are part of the innate immunity systems against infection resulting from pathogens. The cationic domains of these peptides interact with the negatively charged surface of the microorganisms eliciting an antimicrobial reaction when the peptide is inserted into the microbial membranes [37]. Antimicrobial peptides, due to their diversity, elicit antimicrobial activity through other mechanisms such as the Shai-Matsuzaki-Huang, albeit at micromolar concentration [38]. These peptides and other synthetic peptide can be radiolabeled with specificity for detecting localized infections using a variety of chemistries [39]. In this study, by targeting the membrane lipids enriched in bacterial cells, namely PE and Lys-PG, the iminoboronate chemistry allows selective labeling of bacterial cells over mammalian cells [26]. Radiolabeling of AB1-HLys-DOTA and HLys-DOTA peptides with ^64^Cu allowed monitoring of the infection up to 24 h and observation of their biological clearance. This was evident in the superior uptake of ^64^Cu-AB1-HLys-DOTA conjugate in *Staphylococcus aureus* bacteria in comparison to ^64^Cu-HLys-DOTA. Specific bacterial cell uptake of ^64^Cu-AB1-HLys-DOTA was also confirmed by the low uptake (less than 10%) in mammalian SKBR3 breast cancer cells, while specificity in Gram-positive bacteria was confirmed with lower uptake in *Pseudomonas aeruginosa*. Previous studies have shown specific uptake of ^68^Ga-radiolabeled probes at sites of infection [17]. Similarly, in this study, the biodistribution numbers show that ^64^Cu-AB1-HLys-DOTA has higher affinity to the infection and persists within the infection longer than ^64^Cu-HLys-DOTA that lacks the AB1 group.

The difference in accumulation of the peptides shows that the noncovalent attraction of HLys for bacterial cell surface was greatly enhanced by the covalent binding of AB1 to the lipids on the surface. In the biodistribution and PET images, clear uptake of ^64^Cu-AB1-HLys-DOTA was observed at 1 h post injection, which may be due in part to its higher bioavailability (higher %ID/g in the blood) and slower clearance.

^64^Cu-AB1-HLys-DOTA was able to distinguish between Gram-positive bacterial infection and Gram-negative bacterial infection. This class of peptides offers selective synthetic targets for bacterial lipids, which may give rise to new imaging methods of bacterial infection [26]. Although we are yet to ascertain its utility in distinguishing infection from sterile inflammation, but as reported by Sellmyer et al., it is possible to distinguish infection from background and other noninfection inflammation sites. ^18^F-labeled small-molecule antibiotic trimethoprim [^18^F]FPTMP showed high uptake at the infection and low background signal in normal tissues and other noninfection inflammation sites but did not differentiate between Gram-negative and Gram-positive strains [40]. Thus, future effort in work would focus on using the peptide to distinguish sterile inflammation from bacterial infection. Additionally, optimizing the peptides with functional groups that can modulate their clearance rates and therefore increase uptake at the infection sites would be implemented in future work.

## 5. Conclusion

The purpose of this study was to investigate the potential of AB1-HLys-DOTA peptide to image and distinguish infection due to Gram-positive bacteria from Gram-negative bacteria with higher specificity than HLys-DOTA peptide. ^64^Cu-AB1-HLys-DOTA showed higher uptake in *S. aureus* bacteria cells in vitro and improved accumulation at the infection site of SA-inoculated mice compared with the noncovalently targeting ^64^Cu-HLys-DOTA. In the small-animal PET images, the dose uptake of ^64^Cu-AB1-HLys-DOTA at the infected site was distinguishable as early as 1 h after administration, indicating its potential for fast detection of infection. These results illustrate that the ^64^Cu-labeled AB1-HLys-DOTA peptide could be used as imaging probe for detection of bacterial infection in vivo with specificity for Gram-positive bacterial infection.

## Data Availability

The cell uptake, biodistribution, and small animal PET/CT data used to support the findings of this study are included within the article.

## Supplementary Materials

Supplementary Materials Supporting Information Figure 1: (a) HPLC chromatogram of HLys-DOTA and (b) LC-MS of HLys-DOTA with *m*/*z* values at 423.7, *m*/*z*+ = 4; 846.4, *m*/*z*+ = 2; 1692.8, *m*/*z*+ = 1; (c) HPLC chromatogram of AB1-HLys-DOTA and (d) LC-MS of AB1-HLys-DOTA with *m*/*z* values at 519.0, *m*/*z*+ = 4; 691.6, *m*/*z*+ = 3; 1037.0, *m*/*z*+ = 2. Supporting Information Table 1: radiolabeling kinetics of AB1-HLys-DOTA with ^64^Cu at different temperature and labeling ratio. Supporting Information Figure 2: HPLC chromatogram showing the radioactive peak of (a) ^64^Cu-AB1-HLys-DOTA and (b) ^64^Cu-HLys-DOTA and UV absorbance at 280 nm of (c) ^64^Cu-AB1-HLys-DOTA and (d) ^64^Cu-HLys-DOTA. Supporting Information Figure 3: small-animal PET/CT images of (a) ^64^Cu-AB1-HLys-DOTA in mice infected on the right thigh muscle with *Pseudomonas aeruginosa*, PA, (b) mean SUV values calculated from PA infected and PBS control muscle, and (c) post-PET biodistribution of ^64^Cu-AB1-HLys-DOTA at 24 h after i.v. injection. Dashed arrow indicates the PBS-control muscle, and solid arrow indicates the infected right muscle (*n* = 4).
