# Supplementary material for: Radiolabeled Cationic Peptides for Targeted Imaging of Infection
Source: Contrast Media Mol Imaging. 2019 Oct 29;2019:3149249. doi: 10.1155/2019/3149249 (PMC7008241; doi:10.1155/2019/3149249)
Supplement: Supplementary Materials — Supporting Information Figure 1: (a) HPLC chromatogram of HLys-DOTA and (b) LC-MS of HLys-DOTA with m/z values at 423.7, m/z+ = 4; 846.4, m/z+ = 2; 1692.8, m/z+ = 1; (c) HPLC chromatogram of AB1-HLys-DOTA and (d) LC-MS of AB1-HLys-DOTA with m/z values at 519.0, m/z+ = 4; 691.6, m/z+ = 3; 1037.0, m/z+ = 2. Supporting Information Table 1: radiolabeling kinetics of AB1-HLys-DOTA with 64Cu at different temperature and labeling ratio. Supporting Information Figure 2: HPLC chromatogram showing the radioactive peak of (a) 64Cu-AB1-HLys-DOTA and (b) 64Cu-HLys-DOTA and UV absorbance at 280 nm of (c) 64Cu-AB1-HLys-DOTA and (d) 64Cu-HLys-DOTA. Supporting Information Figure 3: small-animal PET/CT images of (a) 64Cu-AB1-HLys-DOTA in mice infected on the right thigh muscle with Pseudomonas aeruginosa, PA, (b) mean SUV values calculated from PA infected and PBS control muscle, and (c) post-PET biodistribution of 64Cu-AB1-HLys-DOTA at 24 h after i.v. injection. Dashed arrow indicates the PBS-control muscle, and solid arrow indicates the infected right muscle (n = 4). [file 3149249.f1.docx]

## (a)


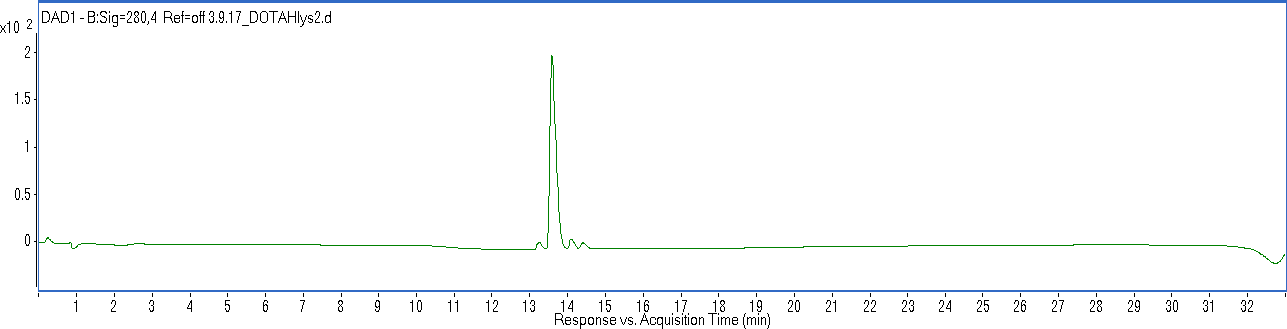

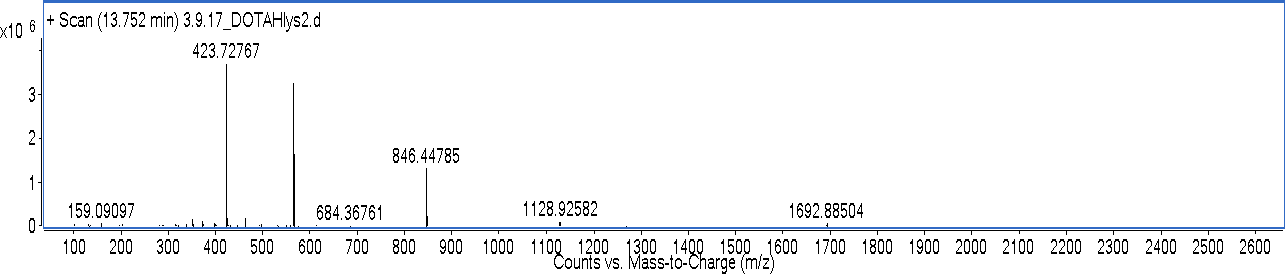

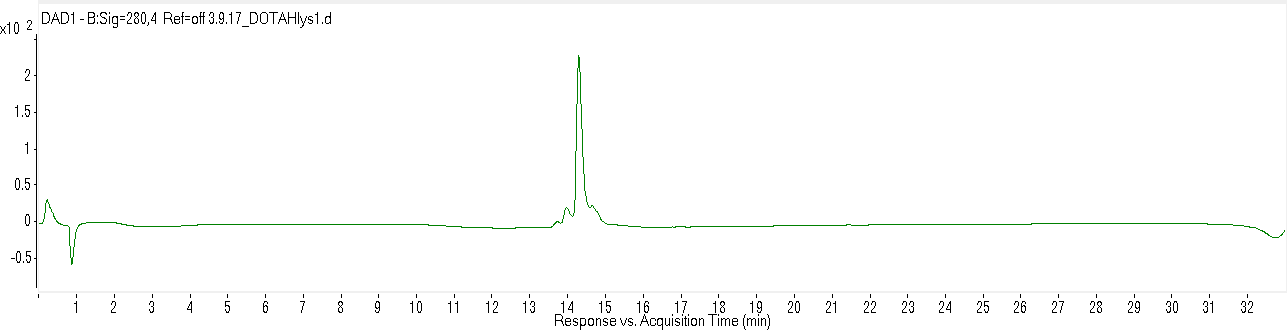

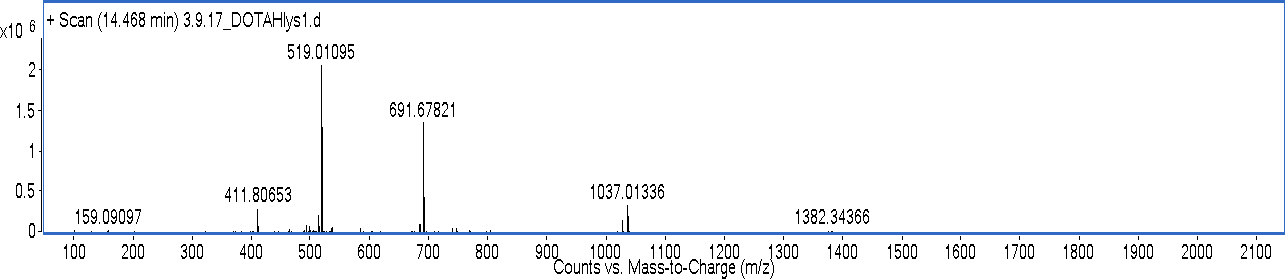


**(b)**

**(c)**

**(d)**

Supporting Information Figure 1: (a) HPLC chromatogram of HLys-DOTA and (b) LC-MS of HLys-DOTA with m/z values at 423.7, m/z+ = 4; 846.4, m/z+ = 2; 1692.8, m/z+ = 1; (c) HPLC chromatogram of AB1-HLys-DOTA and (d) LC-MS of AB1-HLys-DOTA with m/z values at 519.0, m/z+ = 4; 691.6, m/z+ = 3; 1037.0, m/z+ = 2.

Supporting Information Table 1: Radiolabeling kinetics of AB1-HLys-DOTA with ^64^Cu at different temperature and labeling ratio

| **Labeling Ratio, µCi:µg** | **Labeling Temperature** | | |
| --- | --- | --- | --- |
|  | **37°C** | **45°C** | **56°C** |
| 5:1 | 51.5 ± 30.4 | 81 | 76 |
| 10:1 | 55.0 ± 39.6 | 96.5 ± 2.1 | NA |
| 15:1 | 24 | NA | NA |
| 20:1 | 40.7 ± 15.1 | 79 | NA |
| 40:1 | 53.0 ± 7.1 | 69 | NA |
| 70:1 | 42.0 ± 8.5 | 40.5 ± 12.0 | 88.0 ± 7.1 |

**20000**

**15000**

C oun ts

**10000**

**5000**

**5000**

**4000**

**3000**

C oun ts

**2000**

**1000**

### (a )

**0**

**0 2 4 6 8 10 12 14 16 18 20**

T im e ( m in )

### (b )

**0**

**0 2 4 6 8 10 12 14 16 18 20**

T im e ( m in )

**64 Cu - AB1 - HL y s - DO T A - Ra d P e a k 64 Cu - HL y s - DO T A - Ra d P e a k**

**1 20**

**00**

**80**

**60**

**40**

**20**

**0**

**-2 0 2 4 6 8 10 12 14 16 18 20**

**15**

2 80 nm

### (c )

T im e ( m in )

**10**

**5**

A

2 80 nm

**0**

**(d ) -5**

**2 4 6 8 10 12 14 16 18 20**

A

T im e ( m in )

**64 C u -A B 1 -H L y s - D O T A -A ^64^ C u -H L y s -D O T A -A**

**280**

**280**

Supporting Information Figure 2: HPLC chromatogram showing the radioactive peak of (a)

64Cu-AB1-HLys-DOTA and (b) ^64^Cu-HLys-DOTA and UV absorbance at 280nm of (c) ^64^Cu- AB1-HLys-DOTA and (d) ^64^Cu-HLys-DOTA

1h 4h


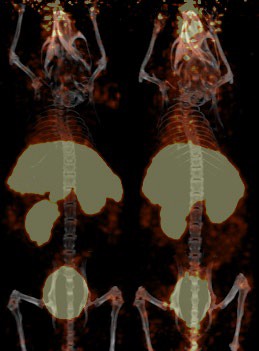

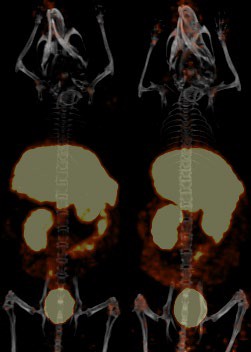

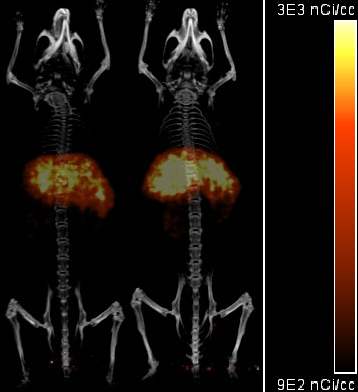


**28**

**0 .5 15**

**0 .4**

**SU V m ea n**

**0 .3**

**2**

**1 .0**

**% ID /g**

**0 .2**

**0 .5**

**0 .1**

**0 .0**

**1h**

**4h 24h P o st P E T**

**0 .0**

A B 1- H Ly s - D O T A _Infec t ion A B 1 - H Ly s - D O T A _C ontrol

# b)

**(c)**

**A B 1- H L y s- D O T A - P A _ 24h p o st P E T**

Supporting Information Figure 3: Small animal PET/CT images of (a) ^64^Cu-AB1-HLys-DOTA in mice infected on the right thigh muscle with *Pseudomonas aeruginosa*, PA, (b) mean SUV values calculated from PA infected and PBS control muscle and (c) Post PET biodistribution of 64Cu-AB1-HLys-DOTA at 24 h after i.v. injection. Dashed arrow indicates the PBS-control muscle and solid arrow indicates the infected right muscle. n= 4.
